# Persistently High Platelet Factor 4 Levels in an Adolescent with Recurrent Late Thrombotic Complications after SARS-CoV-2 mRNA Vaccination

**DOI:** 10.3390/hematolrep16030048

**Published:** 2024-07-29

**Authors:** Yoichi Haga, Akira Ohara, Tsuneyoshi Yakuwa, Akari Yamashita, Midori Udo, Masaki Matsuoka, Hiroshi Ohara, Atsushi Yasumoto, Hiroyuki Takahashi

**Affiliations:** 1 Department of Pediatrics, Toho University Medical Center Omori Hospital, 6-11-1, Omori-Nishi, Ota-ku, Tokyo 143-8541, Japanmasaki.matsuoka@med.toho-u.ac.jp (M.M.); hiroyuki.takahashi@med.toho-u.ac.jp (H.T.); 2 Department of Clinical Laboratory, Toho University Medical Center Omori Hospital, 6-11-1, Omori-Nishi, Ota-ku, Tokyo 143-8541, Japan; 3 Department of Cardiovascular Medicine, Toho University Medical Center Omori Hospital, 6-11-1, Omori-Nishi, Ota-ku, Tokyo 143-8541, Japan; h.ohara@med.toho-u.ac.jp; 4 Department of Laboratory Medicine and Blood Transfusion, Hokkaido University Hospital, North-14, West-5, Kita-ku, Sapporo-shi 060-8648, Hokkaido, Japan; yasuatsu0219@huhp.hokudai.ac.jp

**Keywords:** platelet factor 4, SARS-CoV-2 vaccine, venous thrombosis, antiphospholipid antibody syndrome, anticoagulants

## Abstract

Thrombosis after severe acute respiratory syndrome coronavirus 2 vaccination is a serious complication in patients with a thrombophilic predisposition. Herein, we present a 17-year-old female who had underlying antiphospholipid syndrome (APS) and developed deep vein thrombosis (DVT) 6 months after her second BNT162b2 vaccine dose. Although she had no family history of thrombosis, she had previously developed DVT at 6 years of age, with thrombus formation in the right common iliac vein and the inferior vena cava, along with concomitant left pulmonary infarction. The patient had received anticoagulant therapy for 6 years after DVT onset, with subsequent treatment cessation for 5 years without recurrence. She received the BNT162b2 vaccine at 17 years of age, 1 week before a routine outpatient visit. Platelet factor 4 elevation was detected 14 days after the first vaccination, persisting for 5 months without thrombotic symptoms. Six months after the second vaccine dose, the DVT recurred and was treated with a direct oral anticoagulant. The vaccine was hypothesized to exacerbate the patient’s APS by activating coagulation. Platelet factor 4 levels may indicate coagulation status. When patients predisposed to thrombosis are vaccinated, coagulation status and platelet activation markers should be monitored to prevent DVT development.

## 1. Introduction

The coronavirus disease pandemic caused by severe acute respiratory syndrome coronavirus 2 (SARS-CoV-2) began in 2019. Since then, numerous SARS-CoV-2 vaccines have been developed, and various adverse reactions to vaccination have been reported. Vaccine-induced immune thrombotic thrombocytopenia (VITT), involving antiplatelet factor 4 (PF4)-adenovirus complexes, reportedly occurs within 3 months of adenovirus vector vaccine administration [1]. On the other hand, although VITT after SARS-CoV-2 mRNA vaccine (BNT162b2 vaccine) administration is rare, it is important to predict the possibility of deep vein thrombosis (DVT) development after SARS-CoV-2 vaccination in patients with a thrombophilic predisposition.

Elevated plasma β-thromboglobulin (βTG) and PF4 levels indicate platelet activation; however, PF4 and βTG levels are not routinely measured in clinical practice because they are often released in response to stimuli such as blood sampling.

Nevertheless, thrombus formation and intravascular coagulation due to platelet activation can cause myocardial infarction, nephritis, and the development of pathological conditions. Patients with diabetes mellitus are known to exhibit elevated levels of PF4 and βTG due to concomitant microangiopathy, which can be used to detect DVT. The efficacy of PF4 as a cardiovascular risk biomarker in pediatric patients with chronic kidney disease has also been reported [2], and its importance as a marker of DVT is under evaluation. In addition, PF4 is reportedly elevated in patients with antiphospholipid syndrome (APS) when platelet activity is increased, suggesting that PF4 could be a potential marker for DVT [3].

In the current study, we report a case of pediatric APS in which the PF4 level was persistently elevated after administration of a SARS-CoV-2 mRNA vaccine (BNT162b2), followed by DVT development 6 months after the second vaccination.

Consent for publication was obtained from the patient and her mother. The study protocol was approved by the Ethics Committee of Toho University Omori Medical Center (No. 2022-101).

## 2. Case Presentation

**Figure 1:**
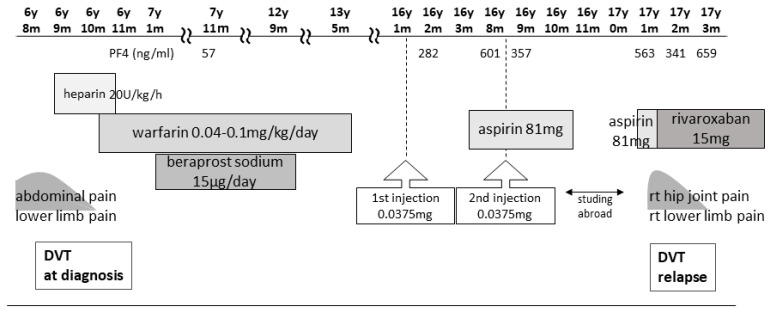
Clinical course from first DVT to second DVT and treatment. DVT, deep vein thrombosis.

The patient was a 17-year-old female with a history of unexplained DVT at 6 years of age, which was diagnosed after the sudden onset of abdominal pain, right groin pain, and vomiting. She had no family history of thrombophilia. Thrombi were detected in the right common iliac vein and the inferior vena cava, with concomitant left pulmonary infarction. Blood tests showed a prothrombin time–international normalized ratio of 1.3, an activated partial thromboplastin time of 37.0 s (reference values: 24.0–39.0 s), a D-dimer level of 88.4 μg/mL (reference values: 0.0–1.0 μg/mL), a fibrin degradation product (FDP) level of 221.6 μg/mL (reference values: 0.0–5.0 μg/mL), a thrombin–antithrombin III complex (TAT) level of 49.1 ng/mL (reference values <3.0 ng/mL), a plasmin-α2 plasmin inhibitor complex (PIC) level of 17.6 μg/mL (reference values: ≤0.8 μg/mL), and a cardiolipin antibody immunoglobulin (Ig)G level of 44 U/mL (reference values: <10 U/mL). A search for causes of thrombophilic predisposition was performed, but no abnormalities were found (Table 1). After 12 days of anticoagulation with unfractionated heparin, the patient was switched to warfarin. Although the pulmonary infarction improved, the thrombi became organized and persisted continuously. Following the DVT diagnosis at 6 years of age, the patient was treated with warfarin for 6 years to prevent recurrence and beraprost sodium (prostaglandin I2 analog) for 5 years. The patient received no antithrombotic therapy for 4 years after warfarin discontinuation, and the thrombosis remained without further DVT for 5 years after cessation of warfarin therapy (Figure 1).

**Figure 2:**
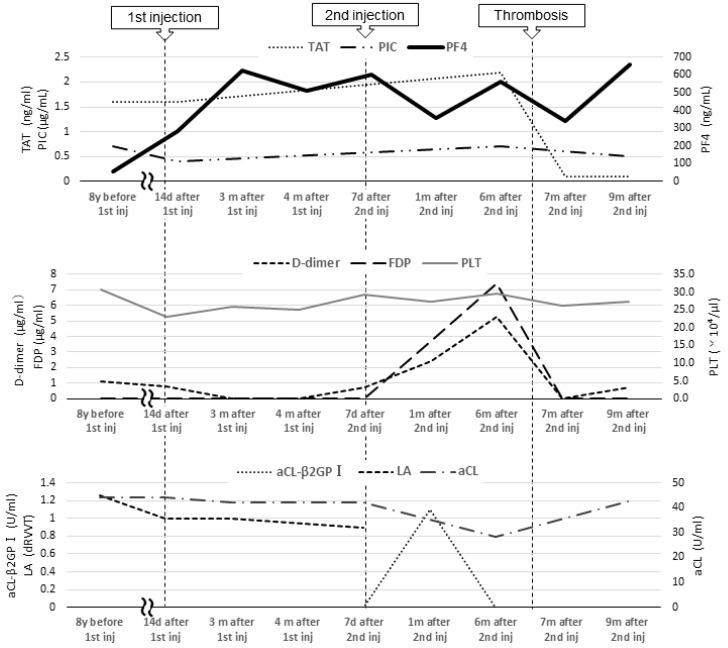
Blood coagulation and fibrinolysis system tests and tests related to antiphospholipid antibody syndrome. Inj, injection; TAT, thrombin–antithrombin III complex; PIC, plasmin-α2 plasmin inhibitor complex; PF4, platelet factor 4; FDP, fibrin degradation product; PLT, platelet; aCL, anti-cardiolipin antibody IgG; aCL-β2GPI, anti-beta-2 glycoprotein I IgG antibody; LA, lupus anticoagulant.

From age 7 to 16, laboratory test results revealed a lupus anticoagulant (LA) level of 1.0–1.26 (reference value: <1.3; assessed using the diluted Russell viper venom time method), an anti-beta-2 glycoprotein I IgG antibody (aβ2GPI-IgG) level < 0.7 U/mL (reference value: <3.5 U/mL), an anti-cardiolipin antibody IgG (aCL) level of 39–44 U/mL, and a moderately elevated aCL level for more than 2 years. At age 16, she was diagnosed with APS based on the 2006 Sapporo criteria by her rheumatologist and pediatrician [4]. The 2023 American College of Rheumatology (ACR)/European League of Rheumatology (EULAR) APS criteria recommend “macrovascular (venous thromboembolism)” and “antiphospholipid antibody (aPL) testing with solid phase-based assay” [5]. At the age of 9, her level of PF4, a marker of platelet activation, was 57 ng/mL (reference values: ≤20 ng/mL). There were no significant changes in activated partial thromboplastin time or LA, aCL, and aβ2GPI-IgG levels at the end of anticoagulation treatment; her PF4 level was not reassessed as thrombosis did not recur. At the time of PF4 measurement, no heparinization was performed (Figure 2).

In 2021, Japan enacted a policy offering the BNT162b2 vaccine to 12- to 17-year-olds. At the age of 16, our patient received her first BNT162b2 vaccine dose one week before her routine outpatient visit (Figure 1). At that time, no distinction was made in Japan between adenovirus vector vaccines and mRNA vaccines, and a causal relationship between all SARS-CoV-2 vaccines and thrombosis was suspected. Owing to her predisposition to develop thrombosis, her PF4 level was measured for the first time in 8 years. Two months post-vaccination, her PF4 level was markedly elevated at 282 ng/mL, increasing to 640 ng/mL after three months; however, as her platelet, FDP, and D-dimer levels were within the reference range, anticoagulants were not prescribed. Because the PF4 level was abnormally elevated post-vaccination, we tested it three times, altering the date, collector, and method of blood collection; the level remained elevated in all test results, ruling out the possibility of a measurement error. The patient was free of any estrogen therapy and was not pregnant. There were no signs of infection such as fever, cough, or diarrhea; no physical findings or blood test results indicative of any infection that could cause thrombosis; and no underlying diseases such as heart disease, diabetes mellitus, or dyslipidemia. No heparin treatment was administered during the PF4 measurement. Consequently, we ruled out causes of inflammation other than APS.

Five months after her first vaccination, the patient was advised to opt out of receiving the second vaccination because of her high PF4 levels. However, because two vaccinations were required as a condition for studying abroad, she opted to receive the second dose. A second vaccination was administered while taking 81 mg of aspirin to suppress platelet activity, and after one month, the PF4 level decreased to 357 ng/mL, and she proceeded to study abroad.

**Figure 3:**
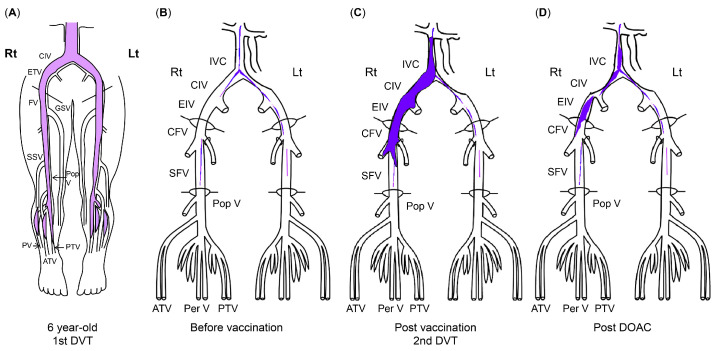
The course of DVT. (**A**) Thrombus at first DVT onset (age 6). (**B**) Organized venous thrombus 1 year before SARS-CoV-2 vaccination. (**C**) Venous thrombus at recurrent DVT diagnosis (11 months after second SARS-CoV-2 vaccination). (**D**) Venous thrombus after DOAC administration. The areas in blue indicate thrombi. ATV, anterior tibial vein; CFV, common femoral vein; CIV, common iliac vein; DVT, deep vein thrombosis; DOAC, direct oral anticoagulant; EIV, external iliac vein; FV, femoral vein; GSV, great saphenous vein; IVC, inferior vena cava; PV/Per V, peroneal vein; Pop V, popliteal vein; PTV, posterior tibial vein; SARS-CoV-2, severe acute respiratory syndrome coronavirus 2.

**Figure 4:**
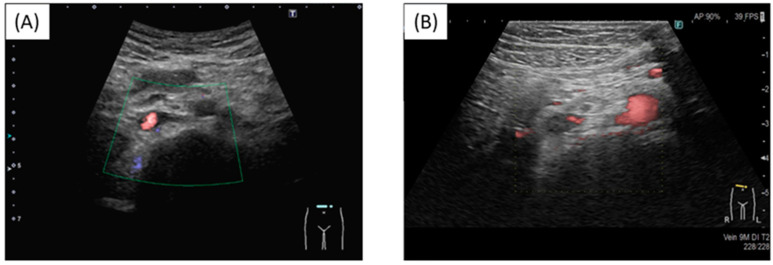
Thrombus in the inferior vena cava. (**A**) Before injection; (**B**) Post injection. New thrombus in the inferior vena cava and reduced blood flow.

At a routine visit 2 months after returning to Japan from studying abroad (6 months after the second vaccination), the patient’s blood tests revealed a normal platelet count and coagulation parameters but elevated PF4 levels (540 ng/mL). Aspirin (81 mg) was administered as an antiplatelet agent; on the fourth day of antiplatelet therapy, she developed chest pain, hypoxemia, and pain and edema in her right lower leg. Her blood test results showed a TAT level of 2.2 ng/mL, a PIC level of 0.7 μg/mL, an FDP level of 7.4 μg/mL, a D-dimer level of 5.3 μg/mL, a PF4 level of 563 ng/mL, and an aCL level of 28.2 U/mL. Abdominal ultrasonography detected an enhancement in the thrombus in the right common iliac vein and inferior vena cava, and a diagnosis of recurrent DVT was established (Figure 3 and Figure 4). The patient’s symptoms and laboratory values improved 3 days after initiating direct oral anticoagulant (DOAC; rivaroxaban) administration. Lung single-photon emission computed tomography (99mTc-macroaggregated albumin, 81 mKr) revealed no pulmonary infarction.

Blood tests 7 months after the second vaccination showed PF4 levels of 341 ng/mL and β-thromboglobulin (βTG) levels as high as 845 ng/mL (reference value: ≤50 ng/mL), but TAT and D-dimer levels were below detection. The SARS-CoV-2 S protein antibody titer was 7610 U/mL (reference value: <0.80 U/mL). Because this case of DVT occurred more than 3 months after the second BNT162b2 vaccination, the anti-PF4/heparin IgG ELISA test could not be performed because the likelihood of DVT was considered low.

Nine months after the second vaccination, her vaccine antibody level was 7230 U/mL, and her PF4 level (not related to vaccine antibody level) remained elevated (659 ng/mL). Currently, her thrombosis is being controlled with rivaroxaban.

## 3. Discussion

This report presents the case of a patient with pediatric APS who developed DVT 6 months after receiving the second dose of the BNT162b2 SARS-CoV-2 vaccine and 11 months after her first vaccination. The persistence of high PF4 levels from vaccine administration to DVT recurrence suggested the occurrence of vaccine-related APS exacerbation.

PF4 is released when tissue factor is overexpressed due to vascular endothelial cell injury or monocyte activation, which activates platelets and promotes platelet aggregation. PF4 levels tend to be elevated when platelet activity is triggered by avascularization or infection. In the current case, the reproducibility of consistently high PF4 levels after BNT162b2 vaccination and the recurrence of DVT when the PF4 levels were elevated suggests a potential relationship between APS and the BNT162b2 vaccine, indicating that the test results for vaccinated patients at risk of thrombosis should be evaluated with caution. In relation to the measurement of PF4 and β-TG, the literature reports the effect of heparin on the mobilization of PF4 from binding sites on endothelial cells, falsely increasing PF4 levels [6]. However, this case is not heparinized.

One differential diagnosis of APS is VITT. SARS-CoV-2 vaccine-associated VITT has been proposed to occur 4–30 days post-vaccination [7]. The most common causative vaccine is ChAdOx1 nCoV-19 [1], with few reports of DVT caused by the BNT162b2 vaccine [8,9]. Although mRNA vaccines are expected to induce thrombosis via a mechanism different from adenoviral vector vaccines, the mechanism underlying mRNA vaccine-associated DVT remains unclear. Several large trials have revealed that mRNA vaccines do not increase the risk of DVT [10]. However, it has recently been reported that mRNA vaccines also induce transient platelet activity, and further mechanisms will be elucidated in the future [11].

In the present case, our patient developed DVT 6 months after the second dose of the BNT162b2 vaccine (11 months after the first dose), and her disease course was distinct from that of VITT. The difference between DVT in this case and VITT is speculated to be related to APS.

APS is an autoimmune disease in which the production of aPLs causes arteriovenous thrombosis. Pediatric APS, defined as APS that develops in patients under 18 years of age [12], is rare, making up only 2.8% of all patients with APS [13]. In the current case, we speculated that BNT162b2 vaccination caused the second thrombosis that occurred 11 years (age 17) after the first (age 6), given the absence of other recurrences. We hypothesized that the thrombosis occurred because the vaccine exacerbated her APS; exacerbating factors associated with pediatric APS include trauma, surgery, neoplasms, nephrotic syndrome, congenital heart disease, obesity, central venous catheter use, prolonged immobilization, stays in the intensive care unit, burns, and mechanical ventilation [14,15]. Typical risk factors such as malignant disease and tobacco use were not observed in our patient.

In a previous case report, Molina-Rios et al. reported the development of systemic lupus erythematosus and secondary APS in a 42-year-old female 2 weeks after her first BNT162b2 vaccine dose [16]. Although the underlying reason remains unclear, it has been suggested that mRNA vaccines are highly immunogenic and induce marked inflammation, which may promote APS development even in asymptomatic aPL-positive patients [17]. Additionally, studies on SARS-CoV-2 mRNA vaccines have reported elevated aPL levels after SARS-CoV-2 infection [18], suggesting SARS-CoV-2 mRNA vaccine is an exacerbating factor for APS. PF4 forms a complex with β2GPI, which is recognized by anti-β2GPI antibodies, and induces platelet activation in APS, which may indicate the disease activity of APS [3]. We hypothesized that the BNT162b2 mRNA vaccine was highly immunogenic; it induced inflammation, which exacerbated the patient’s APS, resulting in persistent inflammation and prolonged PF4 level elevation. And since the patient’s aCL-β2GPI was transiently elevated after the second vaccination (Figure 2), it is possible that the patient developed DVT due to a further increase in APS activity after the second vaccination.

Following platelet activation, platelet microparticles (MPs), known to have high procoagulant activity, are released in large quantities, which, in turn, induce excessive thrombin production, which can also induce venous thrombosis.

In addition, high levels of platelet MPs, tissue factor MPs, and endothelial MPs have been detected in a group of patients with APS [19,20]. APS activation is thought to activate these MPs.

Thus, high PF levels are used as a marker of platelet activation and arterial thrombosis, but persistently high levels may also induce excessive thrombin production and consequent venous thrombosis due to the release of platelet MPs.

Monitoring platelet activity after SARS-CoV-2 vaccination in pediatric patients with APS, such as in the present case, is necessary to prevent DVT. In pediatric patients with a thrombophilic predisposition, platelet activity, and coagulability should be monitored before and after SARS-CoV-2 mRNA vaccination by assessing PF4, βTG, D-dimer, and TAT levels. Given the utility of PF4 and βTG levels in predicting DVT, they may be useful for predicting DVT post-vaccination. Although there is no established index of thromboprophylaxis using PF4 and βTG after BNT162b2 vaccination, in our case, both levels were 10 times higher than their normal ranges. Accordingly, if PF4 and β-TG levels are 10 times higher than their normal ranges after several blood tests, treatment intervention may be necessary.

Regarding thrombosis prophylaxis after mRNA vaccination in patients with APS, it can be difficult to select between warfarin, DOAC, aspirin, and other drugs. In the current case, DVT was considered to be associated with vaccine-induced APS exacerbation. For primary prophylaxis, aspirin doses of 80–100 mg daily and 3–5 mg/kg/day were employed in adult and pediatric patients, respectively [12]. If a patient develops DVT despite taking aspirin, a switch to DOAC should be considered. Accordingly, as observed in the current case, treatment with the DOAC factor Xa inhibitor may be an option. In a previous case report [18], warfarin was used to prevent thromboembolism, and 81 mg of aspirin was administered to reduce PF4 activity, resulting in PF4 reduction to half its original value; however, the patient developed DVT while taking aspirin, and treatment was subsequently switched to DOAC, which improved clinical symptoms. Some reports suggest that warfarin is preferable to DOAC in patients at high risk of thrombosis, including those with APS [21]. However, the suitability of DOAC therapy for patients experiencing APS exacerbation after mRNA vaccination, as observed in the current case, requires further investigation. In children with APS, platelet activity should be monitored by measuring PF4 and βTG levels before and after vaccination. If these levels are elevated, DVT prophylaxis with low-dose aspirin (in accordance with the thromboprophylaxis for APS) may be recommended until the PF4 level is reduced [12]. Monitoring platelet activity after SARS-CoV2 vaccination in children with APS may not directly prevent DVT, but it can alert clinicians to the need for intervention.

A limitation in this case is that the previous PF4 level measurement was performed 8 years before SARS-Cov2 vaccination and could not be monitored immediately prior to vaccination. However, it should be emphasized that the patient continued to exhibit PF4 levels that were 20–30 times higher than the reference value after vaccination and subsequently developed DVT.

## 4. Conclusions

In conclusion, SARS-CoV-2 vaccination triggers platelet and coagulation activation in some predisposed patients, so careful follow-up with platelet and coagulation activation markers such as PF4, βTG, D-dimer, and TAT is important.

## Figures and Tables

**Table 1:** Thrombogenicity test.

	Data		Range
thrombomodulin	13.5	U/mL	(10.4–23.4)
activated protein C	79	%	(73–142)
protein C antigen	65	%	(62–131)
protein S antigen	150	%	(63–135)
free protein S antigen	129	%	(60–150)
PIVKA-II	<1	μg/mL	<1
antithrombin	120	%	(80–120)
total homocysteine	7.8	nmol/mL	(3.0–14)
SF/FMC	negative	μg/mL	<7

PIVKA-II, protein induced by vitamin K absence or antagonist-II; SF/FMC, soluble fibrin/fibrin monomer complex.

## Data Availability

Data sharing is not applicable as no datasets were generated or analyzed during the current study.
